# An Inversion Disrupting *FAM134B* Is Associated with Sensory Neuropathy in the Border Collie Dog Breed

**DOI:** 10.1534/g3.116.027896

**Published:** 2016-08-15

**Authors:** Oliver P. Forman, Rebekkah J. Hitti, Louise Pettitt, Christopher A. Jenkins, Dennis P. O’Brien, G. Diane Shelton, Luisa De Risio, Rodrigo Gutierrez Quintana, Elsa Beltran, Cathryn Mellersh

**Affiliations:** * Kennel Club Genetics Centre, Animal Health Trust, Newmarket, Suffolk, CB8 7UU, United Kingdom; † College of Veterinary Medicine, University of Missouri, Columbia, Missouri 65211; ‡ Department of Pathology, University of California, San Diego, La Jolla, California 92093-0709; § Department of Neurology, Small Animal Clinic, Animal Health Trust, Newmarket, Suffolk, CB8 7UU, United Kingdom; ** The School of Veterinary Medicine, University of Glasgow, G61 1QH, United Kingdom; †† Department of Clinical Science and Services, Royal Veterinary College, University of London, Hatfield, Hertfordshire, AL9 7TA, United Kingdom

**Keywords:** *FAM134B*, GWAS, canine, genome sequencing, sensory neuropathy

## Abstract

Sensory neuropathy in the Border Collie is a severe neurological disorder caused by the degeneration of sensory and, to a lesser extent, motor nerve cells with clinical signs starting between 2 and 7 months of age. Using a genome-wide association study approach with three cases and 170 breed matched controls, a suggestive locus for sensory neuropathy was identified that was followed up using a genome sequencing approach. An inversion disrupting the candidate gene *FAM134B* was identified. Genotyping of additional cases and controls and RNAseq analysis provided strong evidence that the inversion is causal. Evidence of cryptic splicing resulting in novel exon transcription for *FAM134B* was identified by RNAseq experiments. This investigation demonstrates the identification of a novel sensory neuropathy associated mutation, by mapping using a minimal set of cases and subsequent genome sequencing. Through mutation screening, it should be possible to reduce the frequency of or completely eliminate this debilitating condition from the Border Collie breed population.

Sensory neuropathy (SN) in the Border Collie is an autosomal recessively inherited disease first described in the scientific literature in 1987 (Wheeler 1987), with subsequent cases reported in 2005 (Vermeersch *et al.* 2005; Harkin *et al.* 2005). Clinical signs start between 2 and 7 months of age and include progressive proprioceptive ataxia with intermittent knuckling of the paws, hyperextension of the limbs, and self-mutilation wounds in the distal part of the limbs (Figure 1). Usually, the pelvic limbs are more severely affected than the thoracic limbs. There is decreased or loss of proprioception and nociception in all limbs, and in some cases autonomic signs such as urinary incontinence and, in the later stage, regurgitation has also been reported (Vermeersch *et al.* 2005). Electrophysiological studies show decreased or absent sensory nerve compound action potentials, normal or reduced motor nerve conduction velocities, and normal electromyography in the appendicular muscles.

**Figure 1:**
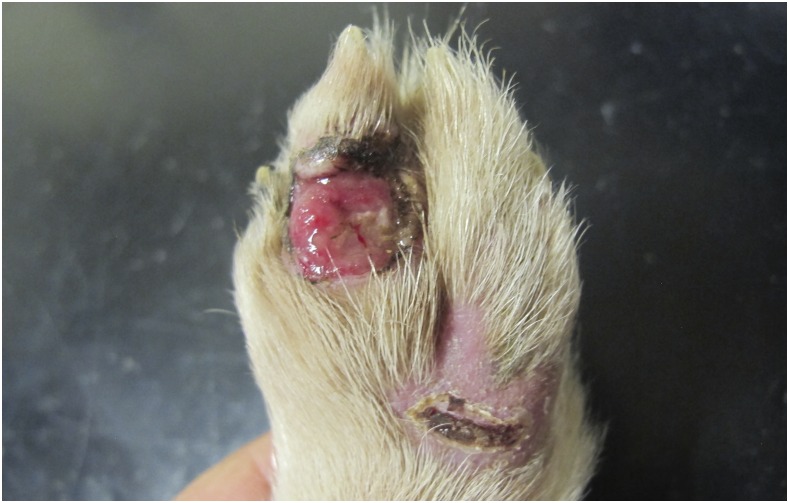
Self-mutilation wound of a sensory neuropathy case. Self-mutilation wounds on the distal part of the pelvic limbs of a 4 month old Border Collie diagnosed with sensory neuropathy.

In analysis of 1 µm resin sections of mixed motor and sensory nerve biopsies from clinically affected Border Collies, the predominant changes in all cases included axonal degeneration, endoneurial fibrosis, and extensive large nerve fiber loss. Results of these studies and illustrations have been previously published (Vermeersch *et al.* 2005; Harkin *et al.* 2005). Involvement of the sensory nerve was consistently severe, while mixed motor and sensory nerves varied from mild to moderate nerve fiber loss. Regenerative clusters or sprouts were not found. Given the severe changes in the sensory nerves and the absence of regeneration, the prognosis for recovery was poor and all reported cases were euthanized within 18 months of diagnosis (Vermeersch *et al.* 2005; Harkin *et al.* 2005).

Although SN in the Border Collie could be categorized as either an inherited sensory and autonomic neuropathy (ISAN) (Granger 2011) or an inherited sensory and motor neuropathy (ISMN) due to the reported motor involvement (Harkin *et al.* 2005), SN in the Border Collie is most comparable with the human hereditary sensory and autonomic neuropathies (HSAN). Dominantly inherited forms include: hereditary sensory and autonomic neuropathy type I (HSAN-I, which is caused by mutations in *SPTLC1*, *SPTLC2*, and *ATL1*; Dawkins *et al.* 2001; Rotthier *et al.* 2010; Guelly *et al.* 2011); Charcot-Marie-Tooth Neuropathy type 2b, which is caused by *RAB7A* mutations (Verhoeven *et al.* 2003); and HSAN-I with dementia and hearing loss, which is caused by mutations in *DNMT1* (Klein *et al.* 2011). Recessive forms include: HSAN-II, which is caused by mutations in *WNK*, *FAM134B*, and *KIF1A* (Shekarabi *et al.* 2008; Kurth *et al.* 2009; Riviere *et al.* 2011); HSAN-III, which is caused by mutations in *IKBKAP* (Slaugenhaupt *et al.* 2001); HSAN-IV, which is caused by mutations in *NTRK1*(Greco *et al.* 1999); and HSAN-V, which is caused by mutations in *NGFB* (Einarsdottir *et al.* 2004). HSAN with spastic paraplegia is caused by mutations in *CCT5* (Bouhouche *et al.* 2006).

Only a modest collection of three SN cases was available for our initial study making a candidate gene study a possible approach. However, suitable breed matched control DNAs were incidentally being genotyped for an independent genome-wide association study (GWAS) so the three SN cases were genotyped in parallel with the available control set, with the aim of identifying potential indicator loci for SN. Genome sequencing would then be used to interrogate suggestive loci from the GWAS for potential causal variants. In summary, our aims were to map the locus for SN using a minimal case set, with the use of genome sequencing techniques to identify the causal variant.

## Materials and Methods

### Diagnosis of SN cases and sample set selection

Three 4-month-old clinically affected [two full-sibling (one male and one female) and one unrelated (one female)] Border Collie dogs were evaluated. The two full sibling dogs belonged to a litter of eight puppies and the third affected dog belonged to another unrelated litter of two puppies. The two full sibling dogs were examined at the Animal Health Trust (AHT) and the third dog at the School of Veterinary Medicine, University of Glasgow.

All three dogs presented with a 2–3 wk history of an insidious onset of chronic progressive proprioceptive ataxia, mainly affecting the pelvic limbs. The unrelated dog also presented with urinary incontinence. Physical examination revealed generalized muscle atrophy mainly affecting the pelvic limbs and self-mutilating wounds on the distal part of the pelvic limbs. The neurological examination revealed normal mental status, and proprioceptive ataxia with spontaneous knuckling more evident on the pelvic limbs. Proprioceptive reactions were absent on the pelvic limbs and decreased to absent on the thoracic limbs. Segmental spinal reflexes were normal. Nociception was absent on the pelvic limbs and decreased to absent on the thoracic limbs. Cranial nerve examination was normal. The cutaneous trunci reflex was present. No discomfort could be detected on palpation of the spine or cranium. Due to the progressive condition and the poor quality of life, the owners elected euthanasia of the three affected dogs. A video demonstrating the clinical signs for sensory neuropathy in the Border Collie is shown in Supplemental Material, File S6.

The two-affected full siblings underwent postmortem examination and peripheral nerve tissue and cerebrum was preserved in RNAlater (Life Technologies) for RNA extraction. Buccal swabs samples were also collected for the extraction of DNA. Sections of the left and/or right sciatic nerve, left and/or right radial nerve, and digital flexor muscles of the thoracic and pelvic limbs were submitted for histopathological studies. The results were consistent with the breed specific (Border Collie) sensory and motor neuropathy (Harkin *et al.* 2005; Vermeersch *et al.* 2005).

The third unrelated dog also underwent postmortem examination and EDTA blood samples were taken for DNA. The histopathological findings in the left sciatic nerve, left common peroneal nerve, and left radial nerve were consistent with the reported SN of the Border Collie. Moreover, there was mild axonal degeneration in the vagus nerve in this third affected dog.

The additional SN cases for mutation screening were clinically and pathologically similar to the three cases used in the initial investigation.

### DNA extraction and genotyping

DNA was extracted from buccal swabs and blood samples using the QIAamp Midi kit (Qiagen). High throughput SNP genotyping was performed using the Illumina CanineHD array, which assays for 173,662 genome-wide SNPs. Genotyping data can be found in File S3, File S4, and File S5. Genotyping data were analyzed using the PLINK software package. Genome-wide homozygosity mapping was performed by allelic association analysis with PLINK (Purcell *et al.* 2007), and only including SNPs that were homozygous in all three cases (85,672 SNPs).

Genotyping of the SN associated inversion was performed by analysis of a fragment length polymorphism generated by PCR. Primers for PCR were as follows: SN_F, 6FAM-TGGAGAACTGACCTGCAACTT; SN_R1, GGCCCGTGTTGTGATCTTAG; SN_R2, AGGGATCATGACCTGAGCTG. PCRs were carried out in 12 μl volumes consisting of 1.5 mM dNTPs, 1× Qiagen PCR buffer, 0.5 μM of each primer, 0.6 U of Qiagen HotStarTaq polymerase, and template DNA. Thermal cycling consisted of 5 min at 95°, followed by 35 cycles of 95° for 30 sec, 57° for 30 sec, and 72° for 30 sec, with a final elongation stage of 72° for 5 min. Products of PCR were analyzed using the fragment analysis module of an ABI3130xl genetic analyzer. Exon resequencing of the *FAM134B* gene was carried out by standard Sanger sequencing methodology, using BigDye3.1 chemistry (Life Technologies) and capillary electrophoresis on an ABI3130xl genetic analyzer. Sequencing data were analyzed using the Staden Gap4 software package. Primer sequences are shown in Table S2 (Staden *et al.* 2000).

### Genome sequencing

Genome sequencing was outsourced to the Wellcome Trust Centre for Human Genetics, University of Oxford. Illumina sequencing of a PCR-free library (100 bp paired-end reads) generated a dataset of approximately 75 Gb, *i.e.*, 31× coverage of the dog genome. Reads were aligned to the canine reference genome (CanFam3.1) using BWA (Li and Durbin 2009) and variant calls made using GATK Haplotype Caller (McKenna *et al.* 2010; DePristo *et al.* 2011; Van der Auwera *et al.* 2013). The genome sequencing dataset can be accessed via the European Nucleotide Archive (ENA accession number: PRJEB12337).

### RNAseq

RNA was extracted from cerebrum using the Qiagen RNeasy Midi kit, and included an on-column DNase treatment. Isolation of mRNA from total RNA was performed using Sera-Mag oligo-dT beads. Libraries for RNAseq were generated using NEBNext Ultra RNA Library Prep Kit for Illumina sequencing. Sequencing was performed on an Illumina MiSeq generating a 4 Gb dataset of 75 bp paired-end reads. Reads were aligned to CanFam3.1 using both Bowtie/TopHat and BWA approaches (Li and Durbin 2009; Kim *et al.* 2013; Langmead 2010). Reads around exon 1 of *FAM134B* were visualized and extracted from the Integrative Genomics Viewer (Thorvaldsdottir *et al.* 2013). Assembly of exon 1 was performed using the Staden Gap4 software package (Staden *et al.* 2000). The RNAseq dataset can be accessed via the European Nucleotide Archive (ENA accession number: PRJEB12352).

### RT-PCR and Sanger sequencing

RT-PCR was carried out using the Qiagen Quantitect reverse transcription kit, followed by PCR with Qiagen HotStarTaq polymerase, using standard reaction conditions. Primers and cycling parameters are listed in File S1.

### Ethics statement

Collection of DNA samples for the GWAS was performed with owner consent by buccal swabbing, which is a noninvasive, nonregulated procedure that does not require a United Kingdom Home Office license. Tissue samples were obtained postmortem after euthanasia of affected dogs on welfare grounds, with full owner consent. Collection of DNA samples from the USA were Animal Care and Use Committee approved, and were collected with informed consent. All other DNA samples were extracted from residual samples taken as part of a veterinary diagnostic procedure, and therefore did not require ethics committee approval.

### Data availability

The genome sequencing dataset can be accessed via the European Nucleotide Archive (ENA accession number: PRJEB12337). The RNAseq dataset can be accessed via the European Nucleotide Archive (ENA accession number: PRJEB12352). The GWAS genotyping data can be found in File S3, File S4, and File S5. The full canine *FAM134B* transcript can be accessed via Genbank accession number KR119069. 

## Results

### Genome-wide association study (SN)

Genome-wide SNP genotyping of three SN cases and 170 controls was performed on the Illumina CanineHD array (173,662 SNPs). All case and control dogs were from the UK. After removing SNPs with a genotyping frequency of less than 95%, 163,524 SNPs remained. No minor allele frequency (MAF) filtering was performed to avoid removal of rare disease associated SNPs. Allelic association analysis revealed multiple strong signals on all chromosomes as a consequence of the small case set (Figure S1). The genomic inflation factor based on the median chi-squared statistic was 1.16. Multidimensional scaling analysis on the N × N matrix of genome-wide identity-by-state pairwise distances, showed the three cases to cluster with the main cluster of controls with no obvious stratification (Figure S2).

**Figure 2:**
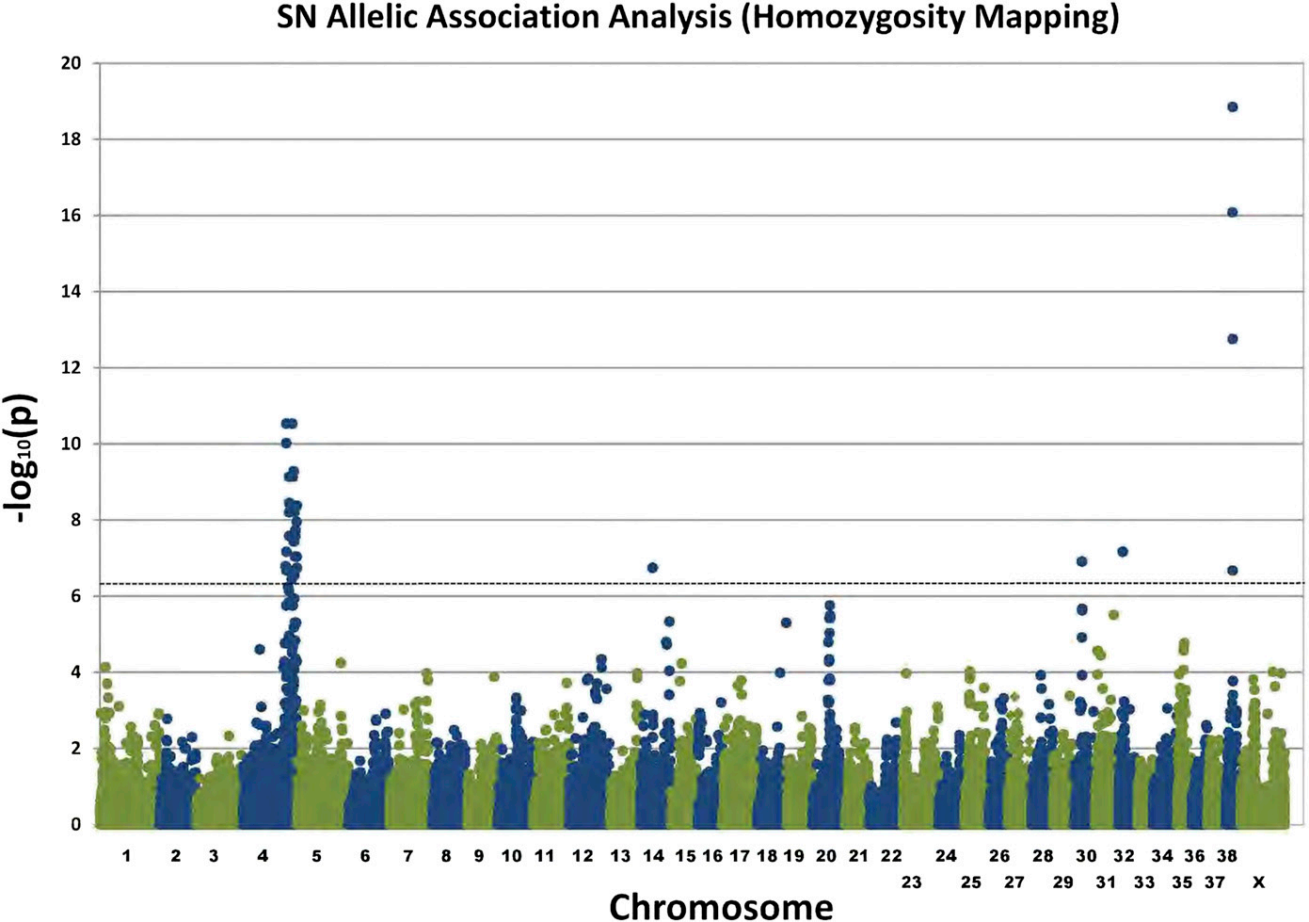
Allelic association analysis plot for the sensory neuropathy (SN) study of three SN cases *vs.* 170 controls. A homozygosity mapping approach was implemented by only using single nucleotide polymorphisms that were homozygous for the same allele in all three cases in the analysis. The dashed line represents Bonferroni significance.

A homozygosity mapping approach was implemented by performing allelic association analysis and only retaining SNPs that were homozygous in all cases (85,672 SNPs). Signals on chromosomes 4, 14, 30, 32, and 38 were defined as genome-wide significant (Bonferroni correction for 86,672 SNPs, *P* < 5.77 × 10^−7^) using the homozygosity mapping approach (Figure 2).

An interval of homozygosity in cases could be defined for the regions on chromosome 4 and 38 as chr4:75,488,014-88,076,462, containing 27 genes and chr38:12,508,392-13,098,194 containing two genes, based on the CanFam3.1 genome build (Table S1). The region on chromosome 4 contained the gene *FAM134B*, which has been associated with autosomal recessive sensory neuropathy (HSAN-II) in humans. Exon resequencing of *FAM134B* in a subset of cases and controls identified no variants that could be considered as potentially causal for SN in the Border Collie. Whole genome resequencing of a single SN case was undertaken to fully investigate the potentially associated chromosomal regions.

**Figure 3:**
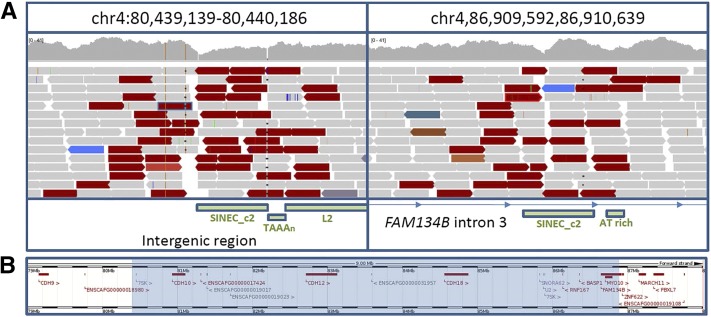
The SN associated inversion. (A) Reads aligning across the inversion breakpoints. Red reads indicate a greater than expected insert size. Read mates for red reads align in the same direction, indicative of an inversion. Repeat elements are shown as green bars with a blue outline. (B) Overview of the genomic region covered by the inversion. The inverted region is highlighted in blue. chr, chromosome; SN, sensory neuropathy.

Due to the strength of *FAM134B* as a candidate, sequence reads aligning across this gene were visually assessed for potential causal variants using the Integrative Genomics Viewer (IGV) (Robinson *et al.* 2011). A 6.47 Mb inversion was identified with breakpoints in intron 3 of *FAM134B* (chr4:86,910,352) and in an upstream intergenic region (chr4:80,439,639) (Figure 3). Samples in the GWAS set were genotyped for the inversion. All three cases were homozygous for the inversion and all 170 controls homozygous for the reference allele (*P* = 3.15 × 10^−77^).

To further validate the identified inversion, three further SN cases from the UK were genotyped using DNA extracted from archived residual blood samples. Additional genotyping was also performed with five SN cases (all Border Collies) obtained from collaborating laboratories presenting with consistent clinical signs, and 11 additional controls. In the replication set, all eight additional SN cases were homozygous for the inversion, and none of the 11 additional controls (*P* = 7.35 × 10^−6^). Genotyping results are shown in Table 1. Given the extremely strong association of SN with inversion on chromosome 4 and the recessive mode of inheritance, the region on chromosome 38 was excluded.

**Table 1:** Genotyping of an extended Border Collie sample set for the SN associated inversion

	wt/wt	INV/wt	INV/INV
UK SN cases	0	0	6
US SN cases	0	0	2
Danish SN case	0	0	1
Irish SN case	0	0	1
Japanese SN case	0	0	1
US controls*^a^*	5	6	0
UK controls	170	0	0
Totals	175	6	11

Results of genotyping 192 Border Collies for the SN associated inversion. SN, sensory neuropathy; wt, reference allele; INV, the inversion allele; UK, United Kingdom; US, United States.

a Relatives of cases.

### RNAseq analysis

**Figure 4:**
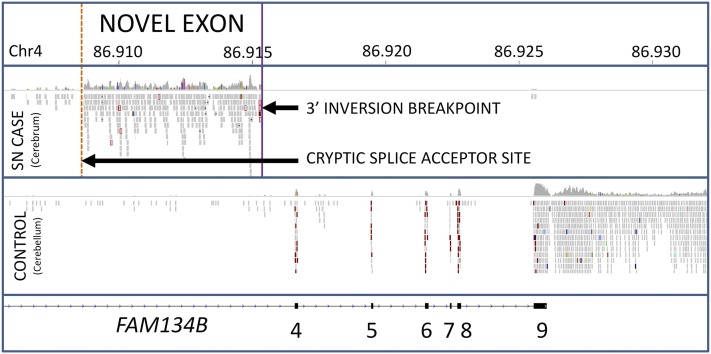
Example of novel exon formation through cryptic splicing. An example of a novel exon occurring before an inversion breakpoint due to a cryptic splicing event. The novel splice acceptor site is located upstream of inversion breakpoint. Two further novel splice acceptor sites are located within the inverted region. Transcription of exons 4 to 9 of *FAM134B* is abolished in the SN case due to relocation of exons 1 to 3 through the inversion event. Note: The control RNAseq dataset is from cerebellum and is shown to illustrate a normal *FAM134B* splicing pattern. The choice of control tissue was based on availability. Chr4, chromosome 4; RNAseq, RNA sequencing; SN, sensory neuropathy

**Figure 5:**
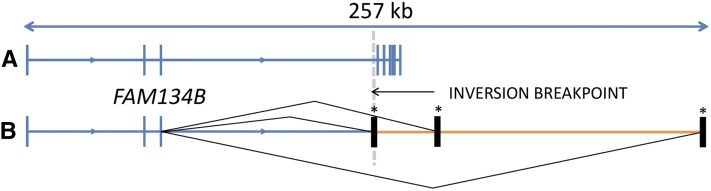
Schematic gene arrangements for the reference and inversion alleles. Transcript arrangements for *FAM134B* for (A) the reference gene arrangement and (B) the gene arrangement after the inversion events. Novel exons are marked with an asterisk, and result in three novel *FAM134B* isoforms. Novel exons all contained stop codons (File S2). The presence of many additional minor novel isoforms cannot be excluded.

To gauge whether the inversion had an impact on the *FAM134B* gene expression pattern, RNAseq was performed. Cerebrum tissue was used based on tissue availability and assessment of expression levels of *FAM134B* by qRT-PCR (data not shown). RNAseq data generated from cerebrum RNA of one SN case demonstrated no detectable expression of *FAM138B* exons situated 3′ of the inversion breakpoint. Expression of novel exons as the result of cryptic splicing was observed after the final normally transcribed exon of *FAM134B* before disruption by the inversion. An example of a novel exon established through a cryptic splicing event is shown in Figure 4. A schematic diagram of *FAM134B* exon arrangements is shown in Figure 5. Novel exons were confirmed by PCR and Sanger sequencing, which also revealed an additional novel exon (File S1). Multispecies alignment of *FAM134B* suggested the Ensembl prediction for canine exon 1 was incorrect. RNAseq data were used to assemble a full *FAM134B* transcript (Genbank accession number KR119069).

## Discussion

In this study, we undertook a GWAS with a minimal number of cases to identify a locus associated with an autosomal recessive disease in the dog. Due to the small number of cases, allelic association analysis using the entire genotyping dataset did not produce a clear genome-wide significant signal, but use of a homozygosity mapping approach enabled a probable disease associated locus to be identified. This study is a further example of how autosomal recessive diseases can be mapped in the dog, even with a very small number of cases, because of the high levels of linkage disequilibrium (LD) within individual breeds (Sutter *et al.* 2004; Safra *et al.* 2013). Despite there being a number of good candidate genes for sensory neuropathy, a GWAS approach was chosen for this study in preference to a candidate gene approach, due to the incidental availability of GWAS data from a large number of suitable controls, with the aim of identifying a novel locus for sensory neuropathy. Given the nonexonic nature of the mutation, the inversion would not have been identified using a candidate gene exon resequencing approach. However, a pattern of linkage disequilibrium for neutral variants in and around the exons of *FAM134B* may have prompted further investigation of this gene. Use of genome sequencing is demonstrated as an effective way of interrogating disease associated intervals, as it provides excellent coverage across all regions including repetitive elements, which are often not captured efficiently using targeted resequencing methods.

Analysis of RNAseq data revealed *FAM134B* was majorly disrupted by the inversion, with novel exons occurring 3′ of the final normally transcribed exon before the inversion, due to cryptic splicing. Although an exact tissue matched control was not available, amplification of two of the three novel exons would not have been possible without the inversion due to primer orientation. The unavailability of suitable antibodies targeting the two genes prevented western blot analysis to determine whether proteins are still produced from the altered transcript sequence. Visual analysis of RNAseq data aligned to the genes flanking and within the inversion region suggests gene expression for these genes was not affected. Genotyping analysis of 192 Border Collies for the SN associated inversion was consistent with the inversion being causal. Based on the location of two identical SINEs (SINEC_c2) at the two inversion breakpoints, we speculate that these repeat elements are likely to have been involved in the inversion mechanism.

The *FAM134B* gene is highly conserved across species and encodes a *cis*-Golgi protein found in sensory and autonomic ganglion neurons (Kurth *et al.* 2009). Little is known about the function of *FAM134B* but it is thought to be critical in the survival of sensory nerve cells (Kurth *et al.* 2009). Knockdown of *FAM134B* has shown to result in structural rearrangements of the *cis*-Golgi compartment, inducing apoptosis in dorsal root ganglia (Kurth *et al.* 2009), highlighting the importance of this gene in the survival of sensory and autonomic neurons. Mutations in *FAM134B* have been associated with SN in humans and, in common with the inversion identified in the dog, all mutations have been high consequence mutations (*i.e.*, nonsense, splice site, and frameshift mutations) (Ilgaz Aydinlar *et al.* 2014; Murphy *et al.* 2012; Kurth *et al.* 2009). This may suggest that major disruption of the gene is required to see the severe early onset phenotype of SN in these cases. Nerve conduction studies in a human patient with *FAM134B* associated SN showed axonal SN with some motor involvement, which is in agreement with the presentation of SN seen in the Border Collie (Murphy *et al.* 2012; Kurth *et al.* 2009). The human *FAM134B* gene codes for a 497 amino acid protein, compared with a 485 amino acid protein in the dog, with the size difference being due to a shorter exon 1 in dog. Exon 1 of canine *FAM134B* is positioned in an unsequenced region of the canine genome, and as a consequence the Ensembl gene prediction for this gene was incorrect. Canine exon 1, which was assembled using RNAseq data, is 82.4% GC rich, which is likely to be reason for its absence from the canine genome build.

In summary, we have used a GWAS approach using a minimal number of cases and genome sequencing to identify an inversion associated with SN in the Border Collie. This approach is particularly attractive when suitable control genotyping datasets from unrelated GWAS are readily available, and has the advantage over candidate gene studies of potential novel locus identification.

## Supplementary Material

Supplemental Material
