# Supplementary material for: An Inversion Disrupting FAM134B Is Associated with Sensory Neuropathy in the Border Collie Dog Breed
Source: G3 (Bethesda). 2016 Aug 15;6(9):2687–92. doi: 10.1534/g3.116.027896 (PMC5015927; doi:10.1534/g3.116.027896)
Supplement: Supplemental Material [file supp_6_9_2687__index.html]

An Inversion Disrupting FAM134B Is Associated with Sensory Neuropathy in the Border Collie Dog Breed — Supplemental Material 

# An Inversion Disrupting *FAM134B* Is Associated with Sensory Neuropathy in the Border Collie Dog Breed

## Supplemental Material for Forman *et al.*, 2016

**Files in this Data Supplement:**

- Figure S1 - Allelic association plot of 3 SN cases versus 170 controls. (.pdf, 4 MB)
- Figure S2 - MDS plot of the 173 dogs genotyped on the Illumina CanineHD array. (.pdf, 110 KB)
- File S1 - Confirmation of novel exons by RT-PCR and Sanger sequencing. (.pdf, 363 KB)
- File S2 - Mutant *FAM134B* transcript isoforms. (.pdf, 277 KB)
- Table S1 - Genes in the disease-associated interval for SN. (.pdf, 26 KB)
- File S3 - CanineHD array genotyping dataset ï¿½ part 1. (.zip, 4 MB)
- File S4 - CanineHD array genotyping dataset ï¿½ part 2. (.zip, 4 MB)
- File S5 - CanineHD array genotyping dataset ï¿½ part 3. (.zip, 5 MB)
- File S6 - A video demonstrating the clinical signs for sensory neuropathy in the Border Collie. (.wmv, 3 MB)
- Table S2 - Primer sequences for *FAM134B* Sanger sequencing. (.xlsx, 12 KB)
