# Supplementary figures and images for: An Inversion Disrupting FAM134B Is Associated with Sensory Neuropathy in the Border Collie Dog Breed
Source: G3 (Bethesda). 2016 Aug 15;6(9):2687–92. doi: 10.1534/g3.116.027896 (PMC5015927; doi:10.1534/g3.116.027896)

# SN Allelic Association Analysis

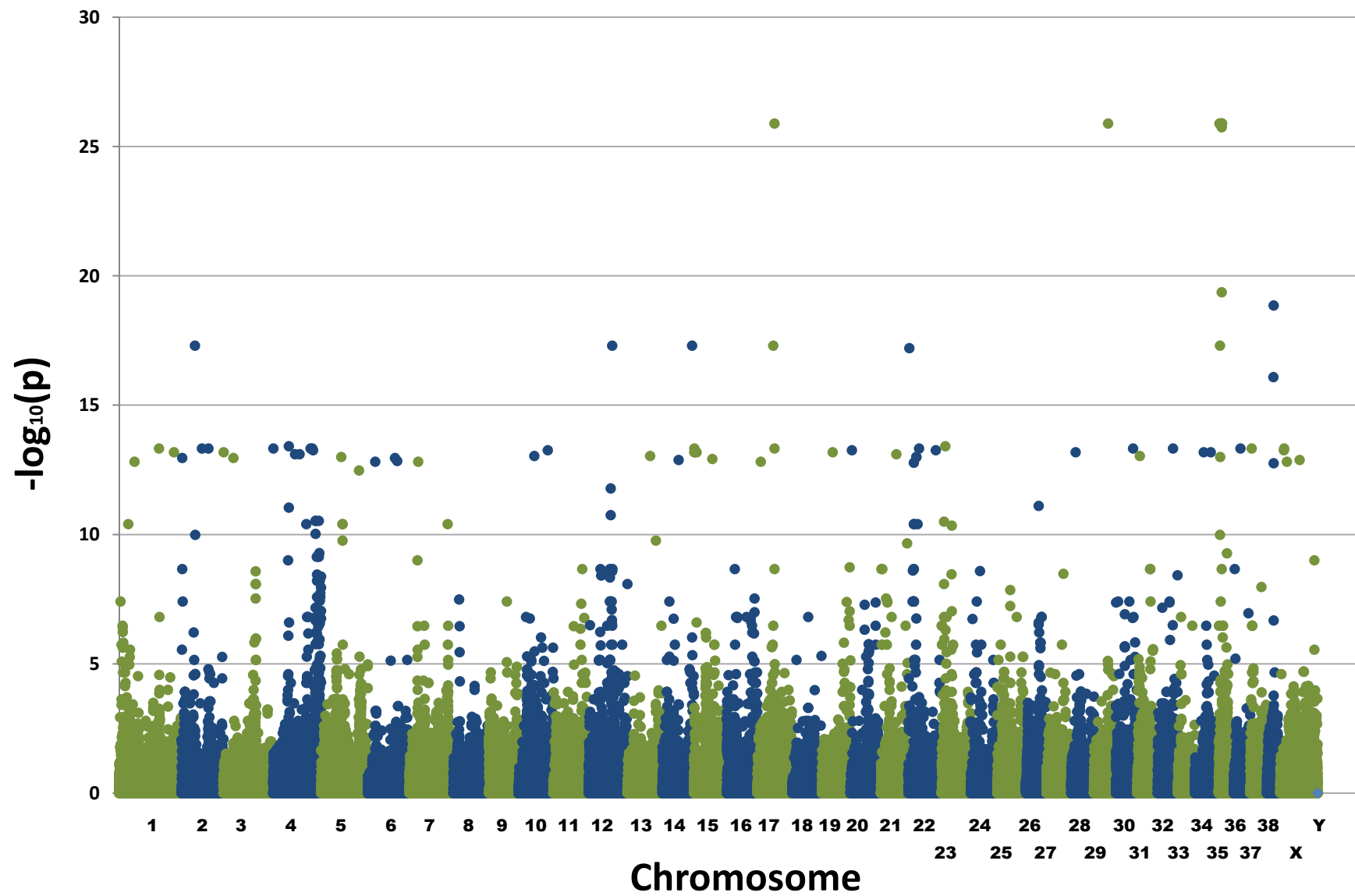

Supplement: Supplemental Material [file supp_g3.116.027896_FigureS1.pdf]

MDS plot - 3 SN cases 170 controls

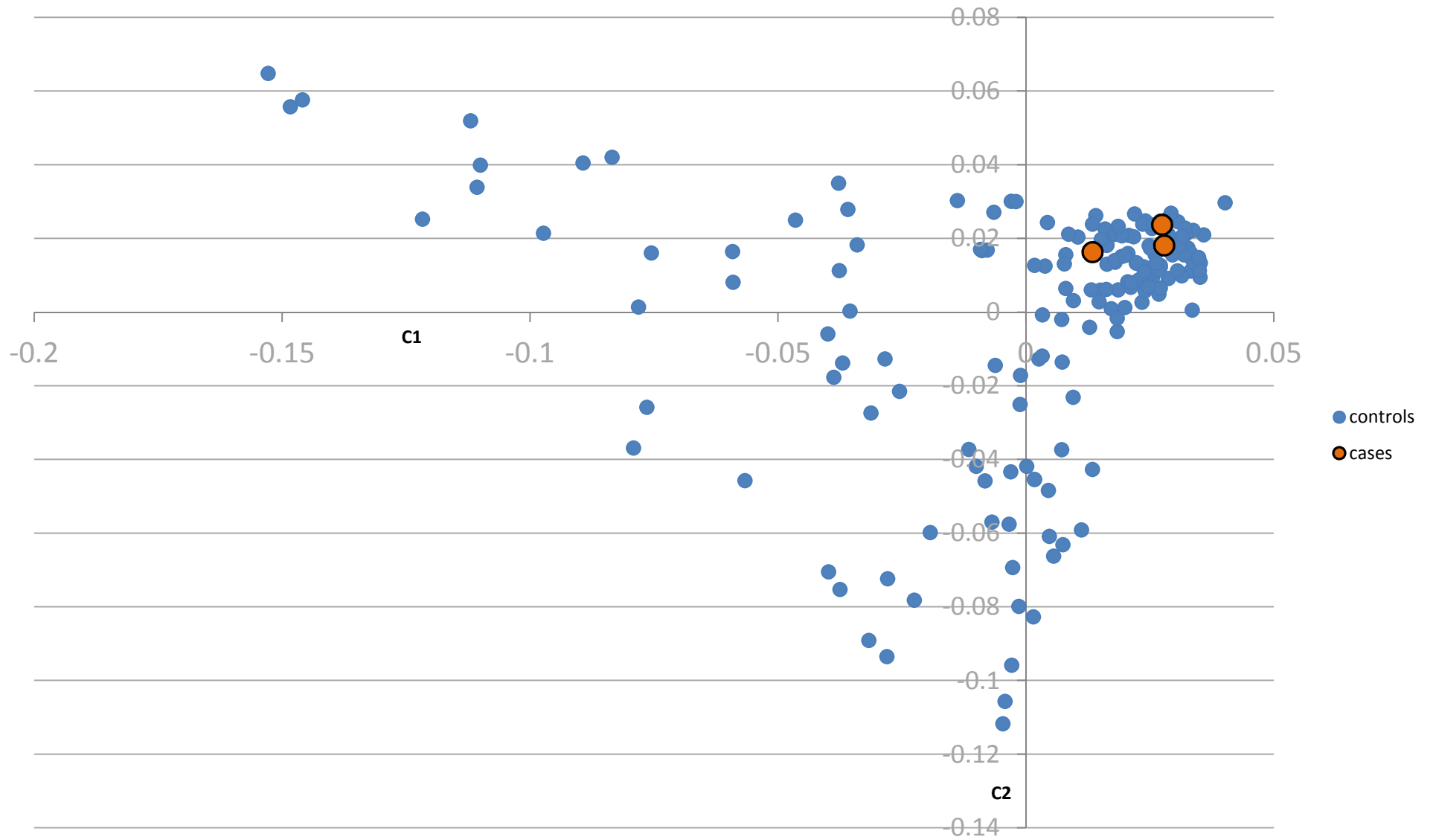

Supplement: Supplemental Material [file supp_g3.116.027896_FigureS2.pdf]
