# Supplementary material for: An Inversion Disrupting FAM134B Is Associated with Sensory Neuropathy in the Border Collie Dog Breed
Source: G3 (Bethesda). 2016 Aug 15;6(9):2687–92. doi: 10.1534/g3.116.027896 (PMC5015927; doi:10.1534/g3.116.027896)
Supplement: Supplemental Material [file supp_g3.116.027896_FileS1.pdf]

RT-PCR amplification of novel isoforms

RT-PCRs were carried out from the final normally transcribed exon to novel exons as indicated by RNAseq data. Sanger sequencing was used to confirm exon-exon boundaries.

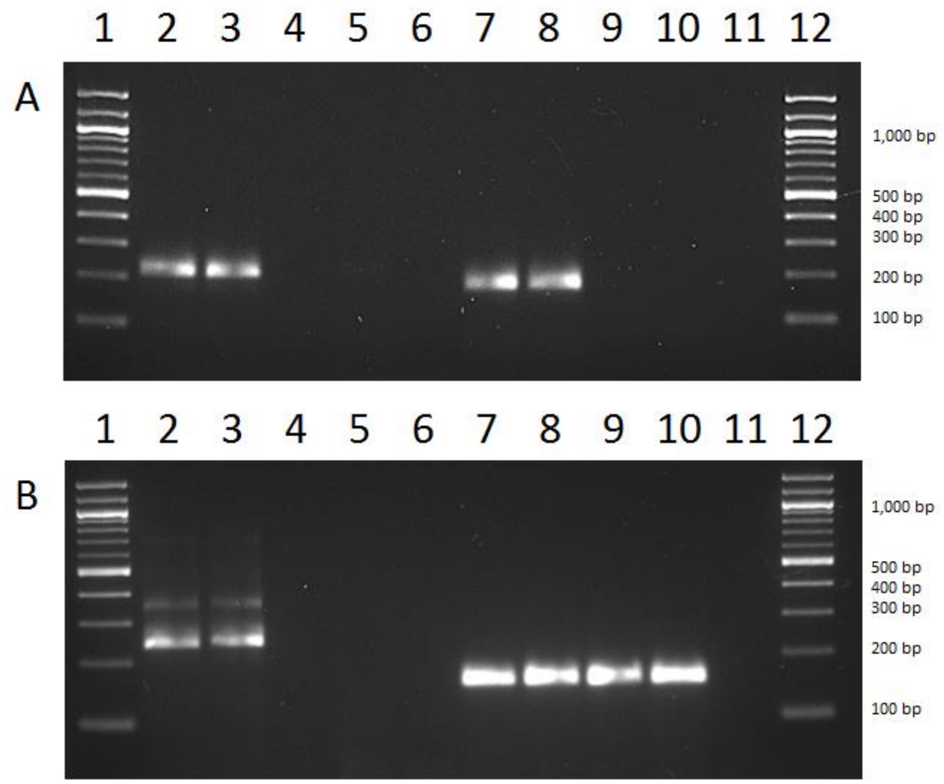

Gel A

1. 100 bp ladder
2. SN case 1 (cerebrum) novel exon RT-PCR 1
3. SN case 2 (cerebrum) novel exon RT-PCR 1
4. Control 1 (cerebellum) novel exon RT-PCR 1
5. Control 2 (cerebellum) novel exon RT-PCR 1
6. NTC novel exon RT-PCR 1
7. SN case 1 (cerebrum) novel exon RT-PCR 2
8. SN case 2 (cerebrum) novel exon RT-PCR 2
9. Control 1 (cerebellum) novel exon RT-PCR 2
10. Control 2 (cerebellum) novel exon RT-PCR 2
11. NTC novel exon RT-PCR 2
12. 100 bp ladder

Gel B

1. 100 bp ladder
2. SN case 1 (cerebrum) novel exon RT-PCR 3*
3. SN case 2 (cerebrum) novel exon RT-PCR 3*
4. Control 1 (cerebellum) novel exon RT-PCR 3
5. Control 2 (cerebellum) novel exon RT-PCR 3
6. NTC novel exon RT-PCR 3
7. SN case 1 (cerebrum) *FBXO34* RT-PCR
8. SN case 2 (cerebrum) *FBXO34* RT-PCR
9. Control 1 (cerebellum) *FBXO34* RT-PCR
10. Control 2 (cerebellum) *FBXO34* RT-PCR
11. NTC TBP RT-PCR
12. 100 bp ladder

* Additional minor novel isoform identified through RT-PCR

Sanger sequence confirmation of novel exons

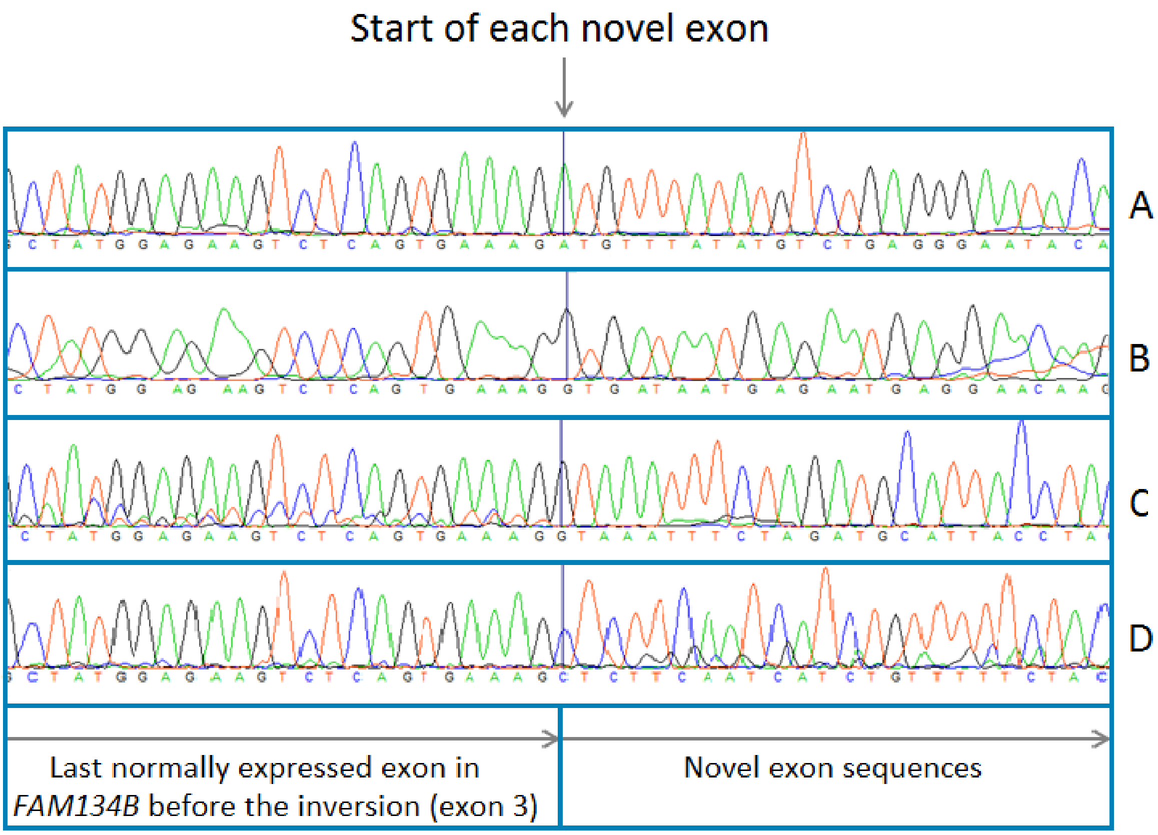

A = SN-FAM134B (Novel Isoform 1)

B = SN-FAM134B (Novel Isoform 2)

C = SN-FAM134B (Novel Isoform 3)

D = SN-FAM134B (Novel Isoform 4)*

Primer Sequences

FAM134B_F	TTGGGCGTGTTATTATGCAA
FAM134B_Novel_R1	CCTTCAAGGCTGAAACATCTG
FAM134B_Novel_R2	GGCATCTTCTTTAGGACCCTCT
FAM134B_Novel_R3	CCCTCTAGATCCATCCATGTTG
FBXO34_F	TTTGAGCCAGATCAGCAAAC
FBXO34_R	GGAGCGAGCTCTCTACTCCT

PCR conditions

95 ˚C 5m

35 cycles:
95 ˚C 30s
60 ˚C 30s
72 ˚C 30s

72 ˚C 5m

12 ˚C Hold
