# Supplementary material for: An Inversion Disrupting FAM134B Is Associated with Sensory Neuropathy in the Border Collie Dog Breed
Source: G3 (Bethesda). 2016 Aug 15;6(9):2687–92. doi: 10.1534/g3.116.027896 (PMC5015927; doi:10.1534/g3.116.027896)
Supplement: Supplemental Material [file supp_g3.116.027896_FileS2.pdf]

Wild-type *FAM134B* and the three SN isoforms formed via cryptic splicing

Canine transcript (Wild-type)

ATGGCGAGCCCGGCGCCCGCGGAGCTCGGCGCGGAGGGATGCCCGGCTCCCGCGGCCGCAGAGCAGGCGCCGCGGGCCCCGCCGCCGCCCCCGG
CCGCCCCCGAGGAGCGCGCGGCGGGCGCGGGGCTGCAGGTGCCGGAGGCGGCGGGCGGGGTGGCGGCCGCGGTGAGCTGGCTGCTCGGGGAGCC
CGGGCTGTGGCTGGGCGGCCGCGCCGACGAGCTCCTGAGCTGGAAGAGGCCGCGGCGCAGCCTGCTCGGCTTCCTGGCGGCCAACCTGCTGTTC
TGGTTCCTCGCGCTGACTCCGTGGAGAGTGTACCACCTGATTTCTGTCATGATACTTGGGCGTGTTATTATGCAAATAATAAAGGATATGGTTT
TGTCTAGAGCTAGAGGTGCCCAGCTGTGGAGAAGTCTCAGTGAAAGCTGGGAAGTTGTCAATTCCAAACCAGATGAAAGACCCAGGTTCAGCCA
CTGTATTGCAGAATCATGGATGAATTTCAGCCTATTTCTGCAAGAAATGTCTCTTTTTAAACAGCAGAGCCCGGGCAAGTTTTGTCTCCTGGTC
TGCAGTGTGTGCACATTTTTTACAATCTTGGGAAGTTACATTCCTGGAGTTATACTCAGCTATCTGCTGTTGCTGTGTGCATTTTTGTGTCCAC
TGTTTAAGTGTAATGATATCGGACAAAAAATATACAGCAAAATCAAGTCAGTTCTGCTGAAGCTAGACTTTGGAATTGGAGAATATATTAATCA
GAAGAAACGTAAGAGATCTGAAGCAGATAAAGAAAAAAGTCACAAAGATGACAGTGAATTAGACCTTTCAGCTCTTTGTCCTAAGATCAGCCTC
ACGGTTGCCGCCAAAGAGCTGTCTGTGTCGGACACAGACGTATCCGAGGTCTCCTGGACTGACAATGGGACCTTCAACCTTTCGGAAGGATACA
CTCCACAGACAGACACTTCTGATGATCTCGATCGACCCAGCGAAGAAGTTTTCTCTCGAGACCTTTCCGATTTTCCATCTGTAGAAAATGGCAT
GGGAACAAATGATGAAGATGAATTCAGCCTTGGCTTGCCCACTGAGCAAAGGAGAAGAAAGGAGCAGCTGGACAGCGGTGTGAGAACGAGCAGA
GAGAGGCAGTCGGCGGCTGGTCTCAGCCTTCCTCTGAGCAGTGACCAAACCTTTCACCTGATGCGCAACCTGGCTGGGGACGCCATCACGGCCG
CAGTGACAGCTGCCATCAAGGAGCAGTTAGAGGGCGCGCAGCAAGCACTCTCTCAGGCTGCCCCCAGCCCAGGAGACGACACAGACACTGAAGA
AGGTGATGACTTTGAACTACTTGACCAGTCAGAGCTTGATCAAATTGAGAGTGAGTTGGGACTCTCACAAGACCAAGAAGCAGAAGCACAGCAA
AATAAGAAGTCTTCGGGCTTCCTTTCAAATCTACTCGGAGGCCATTAG

Canine protein (Wild-type)

MASPAPAELGAEGCPAPAAAEQAPRAPPPPPAAPEERAAGAGLQVPEAAGGVAAAVSWLLGEPGLWLGGRADELLSWKRPRRSLLGFLAANLLF
WFLALTPWRVYHLISVMILGRVIMQIIKDMVLSRARGAQLWRSLSESWEVVNSKPDERPRFSHCIAESWMNFSLFLQEMSLFKQQSPGKFCLLV
CSVCTFFTILGSYIPGVILSYLLLLCAFLCPLFKCNDIGQKIYSKIKSVLLKLDFGIGEYINQKKRKRSEADKEKSHKDDSELDLSALCPKISL
TVAAKELSVSDTDVSEVSWTDNGTFNLSEGYTPQTDTSDDLDRPSEEVFSRDLSDFPSVENGMGTNDEDEFSLGLPTEQRRRKEQLDSGVRTSR
ERQSAAGLSLPLSSDQTFHLMRNLAGDAITAAVTAAIKEQLEGAQQALSQAAPSPGDDTDTEEGDDFELLDQSELDQIESELGLSQDQEAEAQQ
NKKSSGFLSNLLGGH-

SN-FAM134B (Isoform 1)

ATGGCGAGCCCGGCGCCCGCGGAGCTCGGCGCGGAGGGATGCCCGGCTCCCGCGGCCGCAGAGCAGGCGCCGCGGGCCCCGCCGCCGCCCCCGG
CCGCCCCCGAGGAGCGCGCGGCGGGCGCGGGGCTGCAGGTGCCGGAGGCGGCGGGCGGGGTGGCGGCCGCGGTGAGCTGGCTGCTCGGGGAGCC
CGGGCTGTGGCTGGGCGGCCGCGCCGACGAGCTCCTGAGCTGGAAGAGGCCGCGGCGCAGCCTGCTCGGCTTCCTGGCGGCCAACCTGCTGTTC
TGGTTCCTCGCGCTGACTCCGTGGAGAGTGTACCACCTGATTTCTGTCATGATACTTGGGCGTGTTATTATGCAAATAATAAAGGATATGGTTT
TGTCTAGAGCTAGAGGTGCCCAGCTGTGGAGAAGTCTCAGTGAAAGATGTTTATATGTCTGAGGGAATACATGGTGATTATCGCTGATTTGCGA
CGTCCAGAAACACTTGGATAAGTCAACAAGAATCCTGCAGGTGCCCCTAAATAACCACTAAGCCCCTCAGATGTTTCAGCCTTGAAGGCTCTTC
CCGCTGCACGGACTCCGAGCCCCTGCTCCCGGGTCGCGCTCCTGAGCGCCCAGCACCGAGCCTCCCGCCAACGCCTCTAGGAGTCTAGTGGGTC
GGCGGCCCTACAGGCCTGAACTCAGTAGTTGGCGGTGGCAGCCCCAAGAGACTGAGGAGGTGCCGAGGCTGCCAGCGGCGGCTCCCGCCACCCC
GCCTTCCCTTGGGTGACCCGGAGGTGGCTCGGTCCCTGCCTGGCAGGTGCGGCGCTGACGGGGCAGGTGCACAGGCGCGGGGCTGCGAGCCGGG
CCTGGTTGGCACCGCGTGGAGCAGCCGGGGCACAGAGGATGCCAGGCCGCCTGCCTCACTCGCGATCCCCTCCCCTGGAACCCGAGCTGCCCTG
TGGGAGGTTTACACCCGCGGTGGGGGGGCACCTGTGGCTTCCACAGCCTCCCTGCCGCTGCACTGCGTGTCCCCGCCCCGGGAGCCCCCCTGAG
CCCCTGCCCCGGAGCCCTTGCCCCGGGAGCCCCTCCGTGTGCCCCTGCCCCAAGAGCCCCCTGTGCGCCCTTGCCCGGGAGCCCTCTTGCGCCC
CTGCCCTGGGAGCTGCCCCACCCCCACCCACCCCTGCCCCGGGAGCCCCTCTCCCCCCCCACCACGCGCCCCTGCCCGGGAGCCCGGTGCCTGA
AGCACATTCTCTACTCGGTGCCGGTTTATGGACGTGAACGGGAGGGCCGCGGTACAGAGAGGCTACTAGCGCTCTGCCAAGATTCATTTATCCC
GCATCATATTTCCTGGTGCTCAAAGACTCTGGGCGAGAGGGACGAGGCCGGCTGCTAGCACGAGCTCTTACTGAATCACCATCTATTTTTAAAG
AGCACGGGAGAGTCACATTTCCAGTCTTCCAGAACCCCCTGCGGAGCATACCGTGTGCTGCCTTCTGAAGCTCGCTGCCTTCACACTTCATCTC
TGTGGCCGCTGAACTGGACAGAGACAAATTGGACTTTCAATATAAAATTCAGACATTTTTTATTGATATGTTTTGCAAAGAGAGAAGGTACACA
ATAGttttcttaggatgttatgtacttatttctgagagacacagagagagaggcagGCTCCCTGCTGGTGACACCGGGCGTCGTTTTCAACCTC
GGGGTCCCTCGGATGGCTTGGTCCTGGCCTCGGTCCTGGCCCCGGGTGGAGGCCGGTGCGCTGTGGCCTCGACGGGACGGGTCCCCAGCTCCAG
GCTGGCTTCTCTCTGCAGGCTCGGCTCCCGCACGCTCCCCTGCGTCTCCAGCTGCCGTAGCTTCGGTTGGGACCTTGAGTTCTGTGCCCCAGCC
CAGGCGGGCCCTGGTGCAGCGAGCTGCCCTCCCTGGGCTGCGGCCTGCACACCGCAGACGACGGGCCCACCGACGCTCCATCTGCCCGGATAAA
CATCCTTCCTGGCCCAGGATCTTGAAAATCGTTGTTCTGTGTATTCGTCTGCGTTCATGTGTTCGTTCTTTCAGTCTTCCTCTCTCTGTCCCCC
CTCTCCCTCCCCCACTCCGTTCCTTCCCATCTTGTTGTTTCCGATGGAGGGCAGATGAATCCCTGATGTATCATCTTCCCTGACGCCGGTTCAC
CTCTTGTCTCTGTTTTTGAGTCAGGAGCTCTAGCGGCTTCTCCCAGGTGACTCCTTGCAGTGACTCTCTGGGTAAGAAACACCTGCCTCCCAGT
CACTTGGGACCGTCACTGAAAGGGCCGGGTGCTGGGCCTTCCCGCCGGCCTCCCCTGTCGGAGTCTGTGGGGTGAGGCCCAGAAGGCTGCAGTT
CGAGCACCGGGCGGGGGGTTCACACACACTCGATTCGGGAACGGCCCAACGGGCTGGTTTGGCCAGGTCACCAATGTGTGCACTTAATGGTATT
CTAACGGGTAAGGACGCAGGAAGGAGGGAGGTTGGTTTCCAAGCCCACGTGGAAGCCTCGGCTCCCTTCTGTGATAAAGTGAGGCTGCTTGTCT
GAGGAACCATCTTCCTCATTTTTATTCCCCTCCGACTGTCACCGGCAGGTGAAACCTCACCTTCTGCAGCTCCTGACAAGGGAATAACTGCTGA

CCCTGACCATGCAGAGGGTCCCTTGCTAAAAGTCTGTCCCTTAGCTTTTGTTAAGAAGAAGATGGTAACTGAATGGGATCTGCAAGGCCTTAGT
TCTGGTGGCTTTTTCTCGAAAGCACCGCACGTGTGCAATCCCGGGGGGAGCCCTTCGGAGAGCCGGGGGGGAGGGGCCGGGAGTCGTGAGCTTG
AACTGCATTCACCAGGAGTGTGAGAAGGCGGTGATAACGCTGGGTGGGTCGTGAGGGAGCGACTCTTAACTGGCCCTTACCTGTGGCTGGAGTG
CACCGAGGCTCCCTCCTTCGTGAGGGCGAGGGACAGACAAAGCTGGAGCAAAGCCGGGTGCGGGGTAGGGTGGGGACGAGGGACCACAGAACCT
CCCCGGAAAACGCACGTTCTCTCTGAGACTGAAGAGGAAGGACATGAACCCTGGAACATTACACTGAAGCCGTCGGGGATTTTCGCCGTCGGAC
GAGCTTCCATCGTAGAAGACTTACACTCAGACGTCCCGTAAGCCTCCTCTTACATTAGTTGGGCCTCACGGTGCGTGCTGATCCGCGCCAGCGA
TGCTGGGAGGACGCCACACGCCGTGAGAAAGGCCGGTTTCCCCAGCATCGTGCGGTGTTCACAGGGATCCCGTCTCTGGTTATGCGTTCTCCAG
ACGGGCCCTCCAGGAAGGCCGAGAGGCCCCCAGCGAGCAGCGCCGTGGCTGTGTGCATGTCACCTGTGCTAAAACCTCAGCCAGCCACGAGGCA
GACCTTAGTTTGTGCCGAGTATTCAGATGCTGTGGGCGCAGCCTTTTTTCCTGGGAAGGGTGGGTCCTCATGATGCCACGTTGGTGTTGGTCAG
TCACACCTCACTGCTCCCGGCTCTGGAAAGGAGAAGGAACATGGAGCGTTCTTTCTTGTCATAAATTATCTAAAGTCTCATCAAAGGAGCTCAT
TGGATTTATCCATTCTTTGAAGAGGGCGCGTGTGTTAGACAACAGGATGACTGGAAACCAGGAGTCTTGGCCTCAGGTCTATGAACAAACTTGG
GTTTCACTTTCTTCATCTACAAAATAAGACTGAAAAGTTGGTATCAAATGTCGTATTTTATTCCAAAGTCCACAACTGTATTAGATACTATTCA
GTTATGTATGCTGCGTGACTCTGTAATGGTAGACTGTATACAGTGGAAGACTGGAGAGCTGTATGTTCTAGGGGGACTCAGAATTAACTCGCTG
GACAGGTTAAAATGATCATGAAGCTCCCAGAGAGTGACTCTTCTGTTTCGTGTTTTGATTTTTGAttttttaagatttatttatttattttagag
agaggcgagcacatgcaagagcatggggggggaggagggagagagagaatctcaggcagactcactccgagtgagacgctcaacacggggctcga
actcccgaccctgagttcaccacctgagctgaaaccaagaatttgatgcttaaccaactacaccacccagatgcccTGGATCCACGTTTCATTT
AAAGAGGTGTAGAGTGGGCTCTCTGCTTTGCTTTAGACTCAGACTTCTTCATATTTCCACTAAATTTATTCAGCACAATGTATGCATTTTTTTC
TCAAAAACATTTACTATTCTTTTTAAACATTTGCTATTCTTTCCAAATCTCATTTGGTTAGATATTTTCATGTTCTACACAATGGATTCCTCGT
TTCTGTTTGTGGTTGTGACAATAATTCAAATATCAATTAGAATTACTTAAGGTTATGGTGTTAGTATAAGTAACCATAGAAGTTATTGTCCTAA
GATACTGTCACATAAGGCTTCTTCTGAAGGGGAGGAATTTAGTATAAATAAGAACGTCCTTGTATGACATGGGAAATATTCACAAGGTGAATAT
CCTCTTGTTTCTTCTTCCCACCAAGTGGTGGTGTCTGTAGACAGAGAGGGACTGTGCTACAGGTATTCAGTTTGTGCTGGTTTGAAATAACAAC
AGTAAACACACTGGGTATAATATGAGACACAAAGGGGGAACTTGATGACTTTTTATTGAATAAATGAATAAGGATGGATAGATACTGAGGTTTC
TTCATAGCCACTTAATCCCACTATGAAAATAGGTGGTCCTTCAATGTAAAACAAACGTTTTGGTTAATTCATTGTGATATGTATTAAGCACCTT
AAATTGTATGAAGACTTCACCATGACGTTATAATTTAGGCAATTTTAGATGATTTAGTATTAGACTAGGCTCCTTGCTGATTTTAGGGCATTTA
CTTACTAGAATAGTGAAGTATGTATACAAATATGTATAAGTTCTGAGATAGGAATCCTAAAGGTAGTATTAGGACAAGGTTTCAGCATCTCTCT
ATATGGTATAGCTTATTTTAAAAACTGTGTTTCATGGTTTAGCTCTCAGAAATACTGTTTGCTTCACAAATAAACTGAGGCAAAAAACTTAACA
TTTGTATATAGAACGTGAAGTGAGATAGACTCATTGAGAATGTTATTAAATGAAAAGCATGATCATACATGTATCATAACAAATTTTTATTTTT
Attttttaaactaaaaatttattcattcatgagacacagagagagagaggcagagacacaggcagagggagaagcaggctccgtaaggagagc
ccaatgtgagactcgatcccgggaccccggggccatgccatgggcagatgttcaactgctgagccacccaggcgtcccAGGAAtttttattttt
tatttaattgatttttaaaaaagattttattaattcgagagaggctgagagaatgtgtggggaggggcagaaggagaagcggactccctgctga
gcagggagccccaagcggggctgaatcccaggaccctggaatcctgattgagccgaaagcagacgctcaaccgcccgagctactgaggcagccc
tcagatgtttaaataaaggccatatgtgtgtatccgtgtgtagctttaagaacagtagtaagtcggtttgcacgtgtccgtcatctaagtcaag
agctagaacgtgaccaggactcaggaggctcccataggcctcgctggtcacattccctacataccccaccccacccctgtcctggagcttggt
ggtggtggttggtttgttgatgttgctttttttgaaatcatttccttgcctaaacagtttgtcggcttagcatgactttgagcttcatgtaaat
gaagtcatagtgtccgtactctgtgatctttgtctttcggtattttgattctgagggtcacatacagtcacggagttcattcatttcccctgcc
atttcatctttcatggcctggacacaactcactttatctgttcattgtcctcttgaAAATACTCCTAAATTTGTTTCAAAATATGAAATGTTCA
TTCATTCCATTTGAATCGCTGAGAGAATGATAAAGCATTCCATAGGAAGTTTTTCATTGATTTGTTAGAATTGCATTTGCCAGACTGTTCAACC
AAATCCTTAAATATACTATTAAATATGCAGAGAGAGCAGACATTAAAGTCCGTATTTATGTCCACTGTATGGCCCCAGTAAATGAACAGTGGCC
TAGCCTAGTCAATTTGCAGTAAGTCCTCAGGAAGGAGGCCACAgggctcccgggtggctccgttagtgaagtgtctgccttcggctcaggtcat
gctctcagggtcctgggatcgagctgcatgtggggcgccccgctcagcagggaacccgcttctccttctccctctgcccctctccccacttatg
cgcacgctctttctctcaaataaatacatttttttaaaGGCTACACTTTAATGATCGTATTATTTCCTTTGCTCCCCTGCGGTTTTCAGCCTCC
TGTACAAAATCTTTTCCCATTAAGCAGACATACTGGCGAGATTTTGTTACAGAGAGTGGCTTCTATCCATGCACTTAGTGTTTCCTCTGGTAGG
TCTGCAGTCGTTCATAAGGTAGGCAAAAGCTAGTGCTAAATTTGGTCGCCAATGGAAACTGGCTTGCTCAGCGATTCAAGAGAGGGGTAGAGTT
CATGGGTACATAAACCACTGTCCGACAGCTAAGCGTCCCCAAAAGTTCATTTCTTAGGATACTCATTCACAAGTGTCTACCTCATGGGATTGCA
TCTACCTGGAGAACTGGTAGGCCAGTGACTCGGCGAGAGCATTTGACTCTGATAGAATAAAGAGGAAGGAACATGAAATTGTTGTTTTTCCCTT
TTTTAATGCTATTGGATTCACTTAAATGTAACTTAAAATTGTATTTAAAAAGGGTATTTAATTATCT

MASPAPAELGAEGCPAPAAAEQAPRAPPPPPAAPEERAAGAGLQVPEAAGGVAAAVSWLL
GEPGLWLGGRADELLSWKRPRRSLLGFLAANLLFWFLALTPWRVYHLISVMILGRVIMQI
IKDMVLSRARGAQLWRSLSERCLYV-GNTW-LSLICDVQKHLDKSTRILQVPLNNH-APQ
MFQP-RLFPLHGLRAPAPGSRS-APSTEPPANASRSLVGRRPYRPELSSWRWQPQETEEV
PRLPAAAPATPPSLG-PGGGSVPAWQVRR-RGRCTGAGLRAGPGWHRVEQPGHRGCQAAC
LTRDPLPWNPSCPVGGLHPRWGGTCGFHSLPAAALRVPAPGAPLSPCPGALAPGAPPCAP
APRAPCAPLPGSPLAPLPWELPHPHPPLPREPLSPPTTRPCPGARCLKHILYSVPVYGRE
REGRGTERLLALCQDSFIPHHISWCSKTLGERDEAGC-HELLLNHHLFLKSTGESHFQSS
RTPCGAYRVLPSEARCLHTSSLWPLNWTETNWTFNIKFRHFLLICFAKREGTQ-FS-DVM
YLFLRDTEREAGSLLVTPGVVFNLGVPRMAWSWPRSWPRVEAGALWPRRDGSPAPGWLLS
AGSAPARSPASPAAVASVGTLSSVPQPRRALVQRAALPGLRPAHRRRRAHRRSICPDKHP
SWPRILKIVVLCIRLRSCVRSFSLPLSVPPLPPPLRSFPSCCFRWRADESLMYHLP-RRF
TSCLCF-VRSSSGFSQVTPCSDSLGKKHLPPSHLGPSLKGPGAGPSRRPPLSESVG-GPE
GCSSSTGRGVHTHSIRERPNGLVWPGHQCVHLMVF-RVRTQEGGRLVSKPTWKPRLPSVI
K-GCLSEEPSSSFLFPSDCHRQVKPHLLQLLTRE-LLTLTMQRVPC-KSVP-LLLRRRW-
LNGICKALVLVAFSRKHRTCAIPGGALRRAGGEGPGVVSLNCIHQECEKAVITLGGS-GS
DS-LALTCGWSAPRLPPS-GRGTDKAGAKPGAG-GGDEGPQNLPGKRTFSLRLKRKDMNP
GTLH-SRRGFSPSDELPS-KTYTQTSRKPPLTLVGPHGAC-SAPAMLGGRHTP-ERPVSP
ASCGVHRDPVSGYAFSRRALQEGREAPSEQRRGCVHVTCAKTSASHEADLSLCRVFRCCG
RSLFSWEGWVLMMPRWCWSVTPHCSRLWKGEGTWSVLSCHKLSKVSSKELIGFIHSLKRA
RVLDNRMTGNQESWPQVYEQTWVSLSSSTK-D-KVGIKCRILFQSPQLY-ILFSYVCCVT
L-W-TVYSGRLESCMF-GDSELTRWTG-NDHEAPRE-LFCFVF-FLIFKIYLFILERGEH
MQEHGGEEGERESQADSLRVRRSTRGSNSRP-VHHLS-NQEFDA-PTTPPRCPGSTFHLK

RCRVGSLLCFRLRLLHISTKFIQHNVCIFFSKTFTILFKHLLFFPNLIWLDIFMFYTMDS
SFLFVVVTIIQISIRIT-GYGVSISNHRSYCPKILSHKASSEGEEFSINKNVLV-HGKYS
QGEYPLVSSSHQVVVSVDREGLCYRYSVCAGLK-QQ-THWV-YETQRGNLMTFY-INE-G
WIDTEVSS-PLNPTMKIGGPSM-NKRFG-FIVICIKHLKLYEDFTMTL-FRQF-MI-Y-T
RLLADFRAFTY-NSEVCIQICISSEIGILKVVLGQGFSISLYGIAYFKNCVSWFSSQKYC
LLHK-TEAKNLTFVYRT-SEIDSLRMLLNEKHDHTCIITNFYFYFFKLKIYSFMRHRERE
AETQAEGEAGSVRRAQCETRSRDPGAMPWADVQLLSHPGVPGIFIFYLIDF-KRFY-FER
G-ENVWGGAEGEADSLLSREPQAGLNPRTLES-LSRKQTLNRPSY-GSPQMFK-RPYVCI
RV-L-EQ--VGLHVSVI-VKS-NVTRTQEAPIGLAGHIPYIPPPHPCPGAWWWWLVC-CC
FF-NHFLA-TVCRLSMTLSFM-MKS-CPYSVIFVFRYFDSEGHIQSRSSFISPAISSFMA
WTQLTLSVHCPLENTPKFVSKYEMFIHSI-IAERMIKHSIGSFSLIC-NCICQTVQPNP-
IYY-ICRESRH-SPYLCPLYGPSK-TVA-PSQFAVSPQEGGHRAPGWLR--SVCLRLRSC
SQGPGIELHVGRPAQQGTRFSFSLCPSPHLCARSFSQINTFF-RLHFNDRIISFAPLRFS
ASCTKSFPIKQTYWRDFVTESGFYPCT-CFLW-VCSRS-GRQKLVLNLVANGNWLAQRFK
RGVEFMGT-TTVRQLSVPKSSFLRILIHKCLPHGIASTWRTGRPVTRREHLTLIE-RGRN
MKLLFFPFLMLLDSLKCNLKLYLKRVFNY

SN-FAM134B (Isoform 2)

ATGGCGAGCCCGGCGCCCGCGGAGCTCGGCGCGGAGGGATGCCCGGCTCCCGCGGCCGCAGAGCAGGCGCCGCGGGCCCCGCCGCCGCCCCCGG
CCGCCCCCGAGGAGCGCGCGGCGGGCGCGGGGCTGCAGGTGCCGGAGGCGGCGGGCGGGGTGGCGGCCGCGGTGAGCTGGCTGCTCGGGGAGCC
CGGGCTGTGGCTGGGCGGCCGCGCCGACGAGCTCCTGAGCTGGAAGAGGCCGCGGCGCAGCCTGCTCGGCTTCCTGGCGGCCAACCTGCTGTTC
TGGTTCCTCGCGCTGACTCCGTGGAGAGTGTACCACCTGATTTCTGTCATGATACTTGGGCGTGTTATTATGCAAATAATAAAGGATATGGTTT
TGTCTAGAGCTAGAGGTGCCCAGCTGTGGAGAAGTCTCAGTGAAAGGTGATAATGAGAATGAGGAACAAGTAGATTTGCAGCAATGAATTTCAA
ATATTTCTAATCCGTGGATCATTTCACTAGAGGGTCCTAAAGAAGATGCCTCAATGTTCTTCTACAGTAAATGTGTGATAAATACTAATTTATG
TGATTTTTTTCCTCCTAAGAAAATACACACATTGATTTGAATTCCTTGAAAGTAGTATTACTTTTGCCTAAATTTATCCAATATGCTACATAAC
CCTGAAGCACAGTTAGACATTTTATAAATATTGTGTGTGTGTTTTAGTCCATATGTAAGAATTTGGAATTCATGAATGATGACCAGAATGCAAT
GTTAATTTTTGAAGGACTTTCAAGTGTTTTATCCACGTAGCTCATAGCCAAAAAATAAGGTATATCTATCTCCTTTGTTTATTAATCTCTGGTT
TTGGGAAATACTAGAGATTGCATGCATGAAACATTGTAAGTGCTGTACGAATAGAAATGTTAGTTACTATATAATTTGTTTTGTGTTAGGTCTA
AGATTATTTATTTGATATCCTGAGCACCTGTCCACACACAGATAATGGAATTTATTCTCTTTAAATGCATGCTTCTTTTCATACTTCTTGGTGA
TAATCCAATTTTATCTAGTAGCAGGCTGAAAAGAATAATGGAAAAACTAAAAATGCTCCCCCCCTTCCTTCTCTACCATAAGCTATTTTTAGAT
CTTTAATGGAATATATAATGCAACCATTGGAAAAGATGATGCCTTGACTCTTTTCACTGAAATATACACTGAAATAAAATTTGTTGAAAGAGAA
TTGTAGAAATATATGATATAGTAAGTCCAAAAATAAATCTGAAATTGCTCTAATCAAAGTCCTTGCTCTTTAAATTTAATGTGTTGGAAGGAAA
AAGTTTTATATCATCAAGGGCTTTTGTAATGACA

MASPAPAELGAEGCPAPAAAEQAPRAPPPPPAAPEERAAGAGLQVPEAAGGVAAAVSWLL
GEPGLWLGGRADELLSWKRPRRSLLGFLAANLLFWFLALTPWRVYHLISVMILGRVIMQI
IKDMVLSRARGAQLWRSLSER---E-GTSRFAAMNFKYF-SVDHFTRGS-RRCLNVLLQ-
MCDKY-FM-FFSS-ENTHIDLNSLKVVLLLPKFIQYAT-P-STVRHFINIVCVF-SICKN
LEFMNDDQNAMLIFEGLSSVLST-LIAKK-GISISFVY-SLVLGNTRDCMHETL-VLYE-
KC-LLYNLFCVRSKIIYLIS-APVHTQIMEFILFKCMLLFILLGDNPILSSSRLKRIMEK
LKMLPPFLLYHKLFLDL-WNI-CNHWKR-CLDSFH-NIH-NKIC-KRIVEIYDIVSPKIN
LKLL-SKSLLFKFNVLEGKSFISSRAFVMT

SN-FAM134B (Isoform 3)

ATGGCGAGCCCGGCGCCCGCGGAGCTCGGCGCGGAGGGATGCCCGGCTCCCGCGGCCGCAGAGCAGGCGCCGCGGGCCCCGCCGCCGCCCCCGG
CCGCCCCCGAGGAGCGCGCGGCGGGCGCGGGGCTGCAGGTGCCGGAGGCGGCGGGCGGGGTGGCGGCCGCGGTGAGCTGGCTGCTCGGGGAGCC
CGGGCTGTGGCTGGGCGGCCGCGCCGACGAGCTCCTGAGCTGGAAGAGGCCGCGGCGCAGCCTGCTCGGCTTCCTGGCGGCCAACCTGCTGTTC
TGGTTCCTCGCGCTGACTCCGTGGAGAGTGTACCACCTGATTTCTGTCATGATACTTGGGCGTGTTATTATGCAAATAATAAAGGATATGGTTT
TGTCTAGAGCTAGAGGTGCCCAGCTGTGGAGAAGTCTCAGTGAAAGGTAAATTTCTAGATGCATTACCTACTTCTTACAGGTAAATTTCTAGGT
ATTATTGGtgatgaatagaaaaagaagatgtggtgtaccatggaatattactgagccataaagaagaatggaatcttgtcttttgcaacaacat
ggatggatctagagggtattatgctaactgcaataagagagaaagacaaatattgcatcatttcatttacatgtgaaatttaagacatgaatga
aaataaatgaaagaaaaaagaaacaaacaaatgaacaacaacagcaacgaaaacaaaaaacactcttaaatacagagaacaaactgatggttgt
cagaggggaggttgatgggaggatgggtgaaataggtgaagggggattaaaagtatacttatcttgatgagcactaaaaaatgtatagaattgt
tgagtcattctgttgtacatctgaaactaatataacactgcatgctaattatacttcaatgaatagaaacCGGAAATAACTTTACTATGAGAAA
GACTGGGGATATTTGTCATTTCTTAGATGACTCCTCCCTTGGCTTTACCAGTCACTGAAAACGTTGTTCCAAGTACTACTACGAAAGCACAGA
CACAGTTATGCCTGTTATTATtatctatcctgcttagagttcattgtacaaatctgcatcatgatgctttttagcattttaatagtaaacaatt
gtcttttaaaatatgacagatgtatttcacataaaatcttacccttgctctttgaccctccagttacacgcatcttagatttcttctgaatgtc
acaggctttgcatgatactttgtgtattttacatcttttttttgtgtgtgtgtccaatgtgcaattttttctTTAGTTGGGTCCATTAATCAACC
TTTGTAGGTTTGTAAGTCTGCTTTCTCTTGAATTCAAATAATTCTCTCTGAACCTGTGAttattttcaagtttttgcatggtaatcccaatatc
tactcttgtggatgatttctattgtctgcttttccctcagtcaacatttgcctttttaagtacctgaatatgttttaatttttttattatttt
cttaattttgatggagtgccaggtatggcatatgaaaaaatatagaggttacatgaagatttatgatgttctcttcctctaaagggagttcatt
tttgtttcatgaaagtagctaaactgacagcagtaaaatacttcaaagctgcatttttgtctctcagctttgtttctgcttcttcctactccac
agatgtttaattgtggtatttcaagtgaaatctttccttcttatttagagttcctcttcttttgtaatttccaaaccacagtttttgtgcagta
gatcttaagtctgccaaatactctttctggcttctcagtttcttagccatgaattttggaattaccaaatatctcatgtgaaagagtagtgtct

aatgtttttacaaacctgagtcttccttaatccttgaagttggctccacaaattctcactgctctagtaagtctgtgatgccttcagtatgtgt
ttgcttgtttttagtaggttgtcttattgggaaacttgttctggaacgttggaaataaactagtctaccattactatgagtgaaGACCCCCAAT
AATATTTTGATGATCAAAATAAATACAGGTTAaattaattaattaattaattaatGCAACAACTTAAAGAAATTCTATTAAATTGTTCAACTTA
TTTGATATAGTCTATATAGATTATCTTCATGCAACTGAAATACTAATAAAGTTTTAGTACATAGGCTGAAAATATAAAATAACACAAGTAGCAT
TTTTCCCCTTAAAGATTTGTTCATACTTCTTGCATCATGACATGTTATAGTATTTCAAAAAATGTAGAACAGGTATTAAGAATGAAACACAGGT
CGATTTATAGCTGCATATTTTTAAGTCACTAAATCTGAATGTAGGTGAAGTAATTATATGATATGCTTAATTAGGATTTTATTATTTACAAAAT
TATTTCATATACATTGTAGTAGTCAGAACTATAACAAGACTCCCTCCATGATAGCCTGGCTTCACACTGATGGACACATCCTACAAAATCCTAC
AGACTGTGATTTTTGGTGGATTTTTTTCAGGAGATTAGACTATGTGATGTGACAcagttgaccttaagaaaaggggattactttattggaccta
acctattcatatgagccttttaaaatcagagattaaaagcacatgaaagatttggcatactatggctggtttaaagatggagggatcacatgtc
aaaggatgtgggcagtctccaggagtgacagccaacaagaaaacagggacttcaatccatctgccacaggaattaaattcttccaacaacctaa
tgagcttagaagcctattttccccaactctgtatgagaacttggccttaatttcaacctgtgttaccctgagtccagaactcagattcattgtg
ccagacttttgagtcacagaactgtgaactaataaattaatgatattttatgtcattaagtgggtgatattggttataatgcagtggaaaacta
aACCATATGTAATCTCAGTTGATGCTTTTATTAAACCTGTGTAACATTATGACAAGAGGTATTGCTATACACAGTAAGGCAAGATTAAATATCT
AATTAGAAATTGTTCAGGATCTTTGTTTTTTCTCTACCAATAGTTTAGTCCATACACCTGGGGCAAAACCTAGTGATTCAGAGAACAGCTATTG
CCTTCTGTTTTATATATGAATTGATTTCTCTGGTCTGATATGCACAATTTCTGATCCAATGAACTGTACTGTGTACCTTTAGATCAACACCTCA
ATTTGGAATCTGTTAAAGTTATATTTTGGTTCCATTGCTGGGCACTATTACCAGCAAACTTAAGACTCTATATATTAAAGTCCATGAGAGTCAT
GTCCTGGTTCCCTAACTAGATTATTGCAGCAGTGAACTCAAAGTGATCCATGGCCATAGTGAGGAAGATGCAGATCCAGAACTAGAAACCAACA
TTTTGGGTGCAAAATAAGATTCTAGTTCCAATGCAATGCGTGTAAAAATACAAATGAACAACACGAATAGATGAAGAATTCAAAATACTACATG
AAATTTTTATAGAAACAGCAAATCAATTCTGAAGTCATTAGAGATATCAGATATATTTGAGCCATTTTTTTGGAAACTTTTCAAAATAAATTTT
TGAAAATACTTCTAAAAAACGCTATATGAAAAAAATAAAAGGAGTTATATGAATAAAACTTTTAGGATGTAAACTTTTAGAATATACTCAGTAT
GAAGCATTTCTCTTCTTACCTTAGCATGGTATAGCAATTTTCTCCATTACCTGGTTACAGTTCCtaatatattttaattaatatttCTCTTTTC
TCCTGCAGATGGATAATTACTGTGTACTTTAAGGACAATATTTAAGAATATTTTTGTCCACACtttttttttaagactttatttatttattcat
gagaggtacagagagaaagagaggcagagacacaagcgaagggagaagcaggctccatgcagggggcccgacatgggactcgattctgggactc
caggatcacaccctgagccaaaggcagacgctcaactgctcagccacccaggcatctcTATACtttttttttttttaaatcaaaacatacttctt
atttaaaatacttatttaaattattaattCTCACAATATCACTGTGACATCACAATATCGCTGGAGCATTCCAAAATATTAATAAAATAGGATA
TAACTTTATAATACTCTTTCCAGTgggatgcctgcgtggctaattcagttaaaccccctaactcttgattttggatcaggtcatgatctcaggat
catgaaaagcagccctgcctctggctctgcactcaagtctgcttgcctctctctctactccttcccctcactctttctctctctaaaataaata
aaatttaaaaaaTAAAACAAAATATTCTTCCTATACAATACACATAAAATAATAATCTTACATAAACCATGTACCTGTGTATTCAAAGAATATT
AGTATCTTTGTTTTCCTAAGAAGCTATTAGTTTTAG

MASPAPAELGAEGCPAPAAAEQAPRAPPPPPAAPEERAAGAGLQVPEAAGGVAAAVSWLL
GEPGLWLGGRADELLSWKRPRRSLLGFLAANLLFWFLALTPWRVYHLISVMILGRVIMQI
IKDMVLSRARGAQLWRSLSER-ISRCITYFLQVNF-VLLVMNRKRRCGVPWNITEP-RRM
ESCLLQQHGWI-RVLC-LQ-ERKTNIASFHLHVKFKT-MKINERKKKQTNEQQQRKQKT
LLNTENKLMVVRGEVDGRMGEIGEGGLKVYLS--ALKNV-NC-VILLYI-N-YNTAC-LY
FNE-KPEITLL-ERLGIFVIS-MTPPLALPVTENVVPSTTTKSTDTVMPVIIIYPA-SSL
YKSAS-CFLAF---TIVF-NMTDVFHIKSYPCSLTLQLHAS-ISSECHRLCMILCVFYIF
FCVCVQCAIFSLVGSINQPL-VCKSAFS-IQIILSEPVIIFKFLHGNPNIYSCG-FLLSA
FSLSQHLPF-VPEYVLIFLLFS-F-WSARYGI-KNIEVT-RFMMFSSSKGSSFLFHESS-
TDSSKILQSCIFVSQLCFCFFLLHRCLIVVFQVKSFLLI-SSSSFVISKPQFLCSRS-VC
QILFLASQFLSHEFWNYQISHVKE-CLMFLQT-VFLNP-SWLHKFSLL--VCDAFSMCLL
VFSRLSYWETCSGTLEIN-STITMSEDPQ-YFDDQNKYRLN-LIN-LMQQLKEILLNCST
YLI-SI-IIFMQLKY--SFST-AENIK-HK-HFSP-RFVHTSCIMTCYSISKNVEQVLRM
KHRSIYSCIFLSH-I-M-VK-LYDMLN-DFIIYKIISYTL--SEL-QDSLHDSLASH-WT
HPTKSYRL-FLVDFFQEIRLCDVTQLTLRKGDYFIGPNLFI-AF-NQRLKAHERFGILWL
V-RWRDHMSKDVGSLQE-QPTRKQGLQSICHRN-ILPTT--A-KPIFPNSV-ELGLNFNL
CYPESRTQIHCARLLSHRTVN--INDILCH-VGDIGYNAVEN-TICNLS-CFY-TCVTL-
QEVLLYTVRQD-ISN-KLFRIFVFSLPIV-SIHLGQNLVIQRTAIAFCFIYELISLV-YA
QFLIQ-TVLCTFRSTPQFGIC-SYILVPLLGTITSKLKTLYIKVHESHVLVP-LDYCSSE
LKVIHGHSEEDADPELETNILGAK-DSSSNAMRVKIQMNNTNR-RIQNTT-NFYRNSKSI
LKSLEISDIFEPFFWKLFKINF-KYF-KTLYEKNKRSYMNKTFRM-TFRIYSV-SISLLT
LAWYSNFLHYLVTVPNIF-LIFLFSPADG-LLCTLRTIFKNIFVHTFFLRLYLFIHERYR
EKERQRHKRREKQAPCRGPDMGLDSGTPGSHPEPKADAQLLSHPGISILFFF-IKTYFLF
KILI-IINSHNITVTSQYRWSIPKY--NRI-LYNTLSSGMPAWLIQLNP-LLILDQVMIS
GS-KAALPLALHSSLLASLSTPSPHSFSL-NK-NLKNKTKYSSYTIHIK--SYINHVPVY
SKNISIFVFLRSY-F-
