# Supplementary material for: An Inversion Disrupting FAM134B Is Associated with Sensory Neuropathy in the Border Collie Dog Breed
Source: G3 (Bethesda). 2016 Aug 15;6(9):2687–92. doi: 10.1534/g3.116.027896 (PMC5015927; doi:10.1534/g3.116.027896)
Supplement: Supplemental Material [file supp_g3.116.027896_TableS1.pdf]

Table S1

Chromosome	Gene Name	Gene Start (bp)	Gene End (bp)	Ensembl Gene ID	Descriptive Name
4	PDZD2	75351194	75564964	ENSCAFG00000023022	PDZ domain containing 2
4	C5orf22	75748607	75770923	ENSCAFG00000018933	chromosome 5 open reading frame 22
4	DROSHA	75771059	75892510	ENSCAFG00000031829	Uncharacterized protein
4	CDH6	75951481	75999226	ENSCAFG00000018951	cadherin 6, type 2, K-cadherin (fetal kidney)
4	RPS24	76184652	76185904	ENSCAFG00000018956	40S ribosomal protein S24
4	U6	77949902	77950005	ENSCAFG00000027810	U6 spliceosomal RNA
4	CDH9	79153375	79282959	ENSCAFG00000018973	cadherin 9, type 2 (T1-cadherin)
4	Uncharacterized gene	79767507	79768103	ENSCAFG00000018980	Uncharacterized protein
4	7SK	80448563	80448898	ENSCAFG00000027206	7SK RNA
4	CDH10	80927243	81101195	ENSCAFG00000018996	cadherin 10, type 2 (T2-cadherin)
4	Uncharacterized gene	81309938	81310782	ENSCAFG00000017424	Uncharacterized protein
4	GPI	81393940	81395611	ENSCAFG00000019017	glucose-6-phosphate isomerase
4	Uncharacterized gene	81704061	81704803	ENSCAFG00000019023	Uncharacterized protein
4	CDH12	82716681	83126350	ENSCAFG00000019028	cadherin 12, type 2 (N-cadherin 2)
4	Uncharacterized gene	83591088	83592052	ENSCAFG00000031957	Uncharacterized protein
4	CDH18	84562564	84873921	ENSCAFG00000019049	cadherin 18, type 2
4	SNORA62	85827627	85827779	ENSCAFG00000022518	Small nucleolar RNA SNORA62/SNORA6 family
4	U2	85881354	85881544	ENSCAFG00000021169	U2 spliceosomal RNA
4	7SK	85896760	85897093	ENSCAFG00000028137	7SK RNA
4	Uncharacterized gene	86137670	86138749	ENSCAFG00000019051	Uncharacterized protein
4	BASP1	86361608	86362306	ENSCAFG00000029396	brain abundant, membrane attached signal protein 1
**4**	**FAM134B**	**86649536**	**86921116**	**ENSCAFG00000019100**	**family with sequence similarity 134, member B**
4	MYO10	86703260	86793553	ENSCAFG00000019081	myosin X
4	ZNF622	86930022	86951636	ENSCAFG00000023607	zinc finger protein 622
4	MARCH11	87150503	87245227	ENSCAFG00000019105	membrane-associated ring finger (C3HC4) 11
4	FBXL7	87348125	87485086	ENSCAFG00000031644	F-box and leucine-rich repeat protein 7
4	SRP19	87759639	87760052	ENSCAFG00000019108	Uncharacterized protein
38	GPATCH2	12681597	12850636	ENSCAFG00000010781	G patch domain containing 2
38	SPATA17	12850695	13058608	ENSCAFG00000010794	spermatogenesis associated 17
